# A Study on the Prevalence and Subtype Diversity of the Intestinal Protist *Blastocystis* sp. in a Gut-Healthy Human Population in the Czech Republic

**DOI:** 10.3389/fcimb.2020.544335

**Published:** 2020-10-06

**Authors:** Zuzana Lhotská, Milan Jirků, Oldřiška Hložková, Kristýna Brožová, Dagmar Jirsová, Christen Rune Stensvold, Martin Kolísko, Kateřina Jirků Pomajbíková

**Affiliations:** ^1^Biology Center, Institute of Parasitology, The Czech Academy of Sciences, České Budějovice, Czechia; ^2^Faculty of Science, University of South Bohemia, České Budějovice, Czechia; ^3^Department of Bacteria, Parasites and Fungi, Statens Serum Institut, Copenhagen, Denmark

**Keywords:** *Blastocystis*, prevalence, Czech Republic, demography, survey, genetic diversity

## Abstract

*Blastocystis* sp. is a common intestinal protist colonizing the human intestine the prevalence of which varies across non-industrialized and industrialized countries. Its role in the human gut ecosystem remains unclear due to persisting gaps in knowledge of epidemiology and factors affecting gut colonization. Here, we aimed to expand the knowledge of the epidemiology of *Blastocystis* sp. in the gut-healthy humans in one of the industrialized European countries, including the distribution of its subtypes, the correlation between its occurrence and several factors such as lifestyle, contact with animals, age, and sex. A total of 288 stool samples were obtained from asymptomatic individuals over the entire age-range and 136 samples from animals with which the volunteers were in frequent contact. All samples were examined in parallel by PCR and xenic *in vitro* culture. *Blastocystis* sp. was detected in samples from both human and non-human hosts. In humans, the overall prevalence was 24% and eight subtypes were found; in animals, the prevalence was 10%, and only five subtypes were detected. A higher incidence of *Blastocystis* sp. was observed in individuals (i) traveling outside Europe, (ii) in frequent contact with livestock, and (iii) over 50 years of age. We found no effect on gender on *Blastocystis* sp. colonization.

**Summary:**

This study provides data on the prevalence and diversity of the gut protist *Blastocystis* sp. and its subtypes in a gut-healthy human population with emphasis on several factors such as contact with animals, lifestyle, age, and gender.

## Introduction

*Blastocystis* sp. is an anaerobic unicellular eukaryotic organism found in the intestine of a wide range of vertebrates (Alfellani et al., 2013a; Cian et al., 2017; Valença-Barbosa et al., 2019) and some invertebrates (Zaman et al., 1993; Yoshikawa et al., 2016). It belongs to the heterogenous group of organisms called Stramenopiles (Silberman et al., 1996). The life cycle of *Blastocystis* sp. includes four major morphological forms: vacuolar, granular, amoeboid, and cyst (Stensvold et al., 2020a); it is likely that only cyst stages are involved in *Blastocystis* sp. transmission (Stensvold and Clark, 2016a).

*Blastocystis* sp. appears to be a very common colonizer of the human gut all around the globe (Stensvold and van der Giezen, 2018). Although the existence of *Blastocystis* sp. was discovered more than a century ago (Alexeieff, 1911), its role in human health and disease, including its part in the gut microbiome is not yet fully understood. *Blastocystis* sp. has been considered as a potential pathogen associated with irritable bowel syndrome [IBS] (Poirier et al., 2012; Nourrisson et al., 2014) and inflammatory bowel diseases [IBD] (Petersen et al., 2013). However, it appears to be more common in the gut of healthy individuals compared to those with gut disease, and it is now by many considered a commensal (Petersen et al., 2013; Scanlan and Stensvold, 2013; Parfrey et al., 2014; Krogsgaard et al., 2015; Rossen et al., 2015; Stensvold and van der Giezen, 2018; Mardani Kataki et al., 2019; Tito et al., 2019). Moreover, *Blastocystis* sp. might be a potentially important factor in modulating the gut microbiota given its positive correlations with higher bacterial richness and diversity (Andersen et al., 2015, 2016; Audebert et al., 2016; Krogsgaard et al., 2018; Nieves-Ramirez et al., 2018).

Although *Blastocystis* sp. has been suggested to form part of a “healthy gut microbiome,” the epidemiological aspects underlying its occurrence in healthy and diseased individuals are still unclear. Some studies even speculate that the presence of *Blastocystis* sp. might be an indicator of intestinal or even general health (Andersen et al., 2016; Chabé et al., 2017).

The prevalence of *Blastocystis* sp. varies considerably between non-industrialized and industrialized countries (Jeremiah and Parija, 2013; Wawrzyniak et al., 2013; Parfrey et al., 2014; Stensvold and Clark, 2016b). In developing countries, *Blastocystis* sp. is, with prevalence up to 100%, a practically obligate finding in some populations (El Safadi et al., 2014; Poulsen et al., 2016; Mohammad et al., 2017; Oliveira-Arbex et al., 2018), whereas the prevalence reported in industrialized countries most commonly ranges between 7 and 50% (Bart et al., 2013; Scanlan et al., 2014, 2016; El Safadi et al., 2016; Seyer et al., 2017). While the frequent occurrence of *Blastocystis* sp. in human populations in developing countries is probably caused by lower hygienic standard and poorer healthcare (Wawrzyniak et al., 2013; El Safadi et al., 2014; Leelayoova et al., 2018), human colonization by *Blastocystis* sp. in industrialized countries (e.g., Europe, US) may be influenced by several factors such as traveling - mainly to tropical and subtropical countries (Bart et al., 2013; El Safadi et al., 2016), contact with animals (Parkar et al., 2010; Cian et al., 2017; Greige et al., 2018, 2019), diet (Parfrey et al., 2014), or drinking chemically untreated water (Krogsgaard et al., 2015; Angelici et al., 2018; Leelayoova et al., 2018).

Due to the extensive genetic diversity in *Blastocystis* sp., it has proved a challenging task to unravel its taxonomy and develop a useful terminology. To date, 22 different subtypes (ST1–ST17; ST21, ST23–ST26) have been acknowledged in birds and mammals (including humans) based on variation across *Blastocystis* sp. small ribosomal subunit of rRNA genes (SSU rDNA) (Stensvold and Clark, 2020); however, it is very likely that other subtypes will be revealed in future (Jiménez et al., 2019; Maloney et al., 2019; Stensvold and Clark, 2020). The current understanding is that for SSU rDNA sequences to belong to separate subtypes they should generally differ by 4% or more.

Ten subtypes have been isolated from human stool (subtypes 1–9 and ST12), however ST1-ST4 have account for more than 90% of all human carriage (Alfellani et al., 2013b; Scanlan and Stensvold, 2013; Stensvold and Clark, 2016b; Stensvold et al., 2020a). Other subtypes (ST5–ST9) are rare in humans and possibly reflect cases of zoonotic transmission (Alfellani et al., 2013a; Clark et al., 2013; Stensvold and Clark, 2016b). While Europeans are typically colonized by the first four subtypes (ST1–ST4) in more or less equal proportions, ST4 is apparently rare in America, North Africa and the Middle East (Alfellani et al., 2013b; Ramírez et al., 2014, 2016; Jiménez et al., 2019).

The conflicting view on *Blastocystis* sp. in health and disease is primarily based on the persisting gaps in the knowledge about its epidemiology, factors affecting host colonization and interaction with the host, both direct and via the gut ecosystem (reviewed in Lukeš et al., 2015; Chabé et al., 2017). Until very recently, there was almost no information on the prevalence and subtype distribution of *Blastocystis* sp. in the healthy human populations across Western countries. Therefore, *Blastocystis* sp. was detected mainly in patients experiencing gut symptoms and seeking medical consultation. Following these observations, a dogma developed that the occurrence of *Blastocystis* sp. might have a link to clinical manifestations of gut inflammation-associated diseases (e.g., Poirier et al., 2012). At present, it appears to be critical to accumulate data on the occurrence of *Blastocystis* sp. (including the distribution of its subtypes) in industrialized countries to fill the fundamental gap in the knowledge in its circulation in general human population in correlations with various epidemiological aspects such as life-style, contact with animals or diet. So far, only a few epidemiological studies focused on *Blastocystis* sp. in healthy human populations have been conducted in European countries (Bart et al., 2013; El Safadi et al., 2016; Seyer et al., 2017; Krogsgaard et al., 2018), but not in the Czech Republic and entire Eastern Europe. The main aim of this study was to determine the prevalence and subtype diversity of the gut protist *Blastocystis* sp. in an asymptomatic volunteer group across the Czech Republic, who do not suffer from any gastrointestinal symptoms or chronic inflammatory bowel diseases. In addition, we investigated correlations between occurrence of *Blastocystis* sp. and several specific factors. In particular, we were interested in contact with animals, whether it can affect the occurrence of *Blastocystis* sp. in humans and whether can be confirmed. Other factors monitored included lifestyle (urban life versus village one, traveling), gender, and age.

## Materials and Methods

### Sample Collection and Study Area

The present study was conducted in the Czech Republic between 2017 and 2019. Stool samples were obtained from healthy participants who volunteered to participate in this study. None of the volunteers experienced gastrointestinal symptoms at the time of sampling (e.g., abdominal pain, diarrhea, flatulence). In addition to human samples, fecal samples from animals, with which these people were in a close contact (sharing household with pets, in daily contact with farm animals), were also collected to investigate the existence of a potential zoonotic reservoir. Various strategies were used to reach participants, such as information posters, newspapers, magazines, and TV shows. Those who were unable to deliver the sample in person sent it by post as instructed. All participants completed a short questionnaire, which included information on lifestyle (village/city life, traveling), contact with animals, gender and age. Each participant also confirmed the absence of gut inflammation-associated diseases (Crohn's disease, ulcerative colitis) in the questionnaire. “Village” was defined by population, this category included small municipalities of up to 2000 inhabitants. “Traveling” was divided into three subcategories: (i) people who never traveled in the past (i.e., never during life before sampling in this study), (ii) people who had traveled within Europe only, and (iii) those who had traveled outside Europe. The category “contact with animals” was divided into two different subgroups: (i) pet animals (dog, cat, etc.) and (ii) farm animals (pig, cow, horse, etc.). “Age categories” were created based on different life periods and divided into eight categories [i.e., (i) 0–3: infancy + toddler age, (ii) 4–6: preschool age, (iii) 7–12: younger school age, (iv) 13–17: adolescence, (v) 18–30: young adult age, (vi) 31–49: active age, (vii) 50–60: middle age, (viii) >60: retirement]. Additionally, the circumstance of two or more individuals living together was defined as a ‘family’. The questionnaire also included the information about the type of diet (i.e., vegetarian versus non-vegetarian), however, we were not able to obtain a sufficient number of samples from vegetarians/vegans. We always obtained the signed informed consent with each human sample.

Due to the difficulty in communication with most volunteers after the sample delivery, we were unable to conduct a follow-up study to determine how long *Blastocystis* sp. colonization persists or whether they have some periods with clinical gastrointestinal symptoms.

### Ethical Approval

Each participant signed an informed consent declaration to participate in the study. The process, conditions and ethical rules of this study adhered to the Declaration of Helsinki 2013 (World Medical Association). All data were strictly anonymized and processed according to valid laws of the Czech Republic (e.g., Act no. 101/2000 Coll and subsequent regulations). The study was approved by the Ethics Committee of the Biology Center of the Czech Academy of Sciences (reference number: 1/2017).

### Sample Processing and Cultivation Diagnostics

All obtained samples were subjected to two diagnostic approaches: (i) *in vitro* cultivation and (ii) molecular diagnostics. For *in vitro* cultivation, approximately 200 mg of each fecal sample was homogenized and inoculated into a cultivation tube (10 ml; Sigma-Aldrich, St. Louis, MO, USA) containing 4 ml of modified Jones medium (Leelayoova et al., 2002; Suresh and Smith, 2004) supplemented with 10% heat-inactivated horse serum (Sigma-Aldrich, St. Louis, MO, USA) and incubated at 37°C in anaerobic conditions for 48 h (Clark and Stensvold, 2016). Next, samples were inoculated into fresh culture medium for further 48 h. All cultures were evaluated for the presence of *Blastocystis* sp. by light microscopy (Olympus CX22LED) using 400× magnification. The remainder of the fecal sample that was not used for culture was stored at −20°C for subsequent molecular analyses.

#### DNA Extraction

Total genomic DNA was extracted directly from fecal samples using the commercial kit PSP Spin Stool DNA Kit (Stratec, Germany) following the manufacturer's protocol. The isolated DNA (total volume−200 μl and concentration 120–850 ng/μl) was aliquoted and kept at −20°C until analyzed.

#### Molecular Detection and Subtyping of *Blastocystis* sp.

PCR amplification of SSU rDNA was performed using barcode primers RD5 (5′-ATCTGGTTGATCCTGCCAGT-3′) and BhRDr (5′-GAGCTTTTTAACTGCAACAACG-3′) that amplify an ~600 bp region containing sufficient information for phylogenetic analysis and subtype identification of *Blastocystis* sp. (Scicluna et al., 2006; Stensvold and Clark, 2016b). PCR was performed in the T100^TM^ Thermal Cycler (Biorad, USA) under the cycling conditions as follows: 94°C/5 min; 34 × (94°C/1 min; 56°C/1 min; 72°C/1 min); 72°C/5 min. All PCR reactions were prepared in a final volume of 10 μl, containing 5 μl of commercially produced 2× concentrated Master Mix (AccuPower® Taq PCR PreMix; Bioneer, Republic of Korea), 1 μl of each primer (2.5 pmol), 2 μl of extracted DNA, and 1 μl of miliQ water. PCR products were then visualized by electrophoresis; loading 8 μl of PCR product on a 1% agarose gel with ethidium bromide (0.002 mg/ml) using the electrophoresis system (Thermo Fisher Scientific, Inc., USA). PCR amplicons of the appropriate size were purified using the GenElute^TM^ Gel Extraction Kit (Sigma-Aldrich, MO, USA) and sequenced in both directions using PCR primers, sequencing was performed by a commercial company (Eurofins GATC Biotech, Germany). In case of ambiguous sequences with multiple signals, amplicons were subjected to cloning using pGEM®-T Easy Vector System I (Promega, USA). All sequences were processed in the software Geneious Prime 2019.0.4. and subsequently compared to sequences in the GenBank ^TM^ database (National Center for Biotechnology Information) using BLASTn. To determine the subtypes, the sequences were typed using the barcoding platform for *Blastocystis* sp. (http://pubmlst.org/blastocystis/) enabling analysis based on SSU rDNA alleles, which is a valid indicator of genetic variability within subtypes (Scicluna et al., 2006; Stensvold et al., 2012).

### Phylogenetic Analysis

Phylogenetic analyses were used to confirm the *Blastocystis* sp. subtype results and to further analyze those sequences that could not be recognized by the barcoding platform. DNA sequences obtained for this study were deposited in GenBank under the accession numbers (MT039538-99, MT042786-824; for details see Supplementary Material 1). A final dataset for phylogenetic analysis was created to represent the main subtypes and also to cover the diversity of *Blastocystis* sp. from different hosts. All sequences were aligned using the software MAFFT (Katoh et al., 2005), and the alignment was manually edited in the software package Geneious Prime 2019.1.1 (https://www.geneious.com). The best model of the nucleotide substitution was selected by a score of the Akaike information criteria in the built-in IQ-TREE software algorithm ModelFinder (Trifinopoulos et al., 2016) and all phylogenetic calculations were performed under the GTR+F+I+G4 model. Phylogenetic analyses were performed using maximum likelihood (ML) and Bayesian inference (BI). Results for ML were generated by the software PHYML (Guindon and Gascuel, 2003) with 1,000 resamplings of the bootstrap method for branching patterns within trees. Bayesian inference runs were performed for 10 million generations in eight chains and four independent runs by the software MrBayes v3.2 (Ronquist et al., 2012). Coherence of each run was checked in the software TRACER v1.7.1 (Rambaut et al., 2018) and the estimated burn-in was 1,2 million generations. Trees were visualized in the software FigTree v1.4.3 (Rambaut, 2016). Sequences of a closely related taxon (*Proteromonas* sp.) were used to root both trees. The untrimmed alignment has been deposited into DRYAD digital repository.

### Statistical Analysis

The chi-square test was used for statistical evaluation of the significance of the difference in *Blastocystis* sp. prevalence between groups of humans within a single observed factor (lifestyle–urban/rural life, traveling, animal contact, gender). Comparisons between prevalence and age groups were performed by Welch Two Sample *t*-test. A *p* value less than 0.05 was considered to indicate statistical significance. Analyses were performed using software GraphPad Prism 5.0 and R Studio version 3.6.2.

## Results

**Figure 1:**
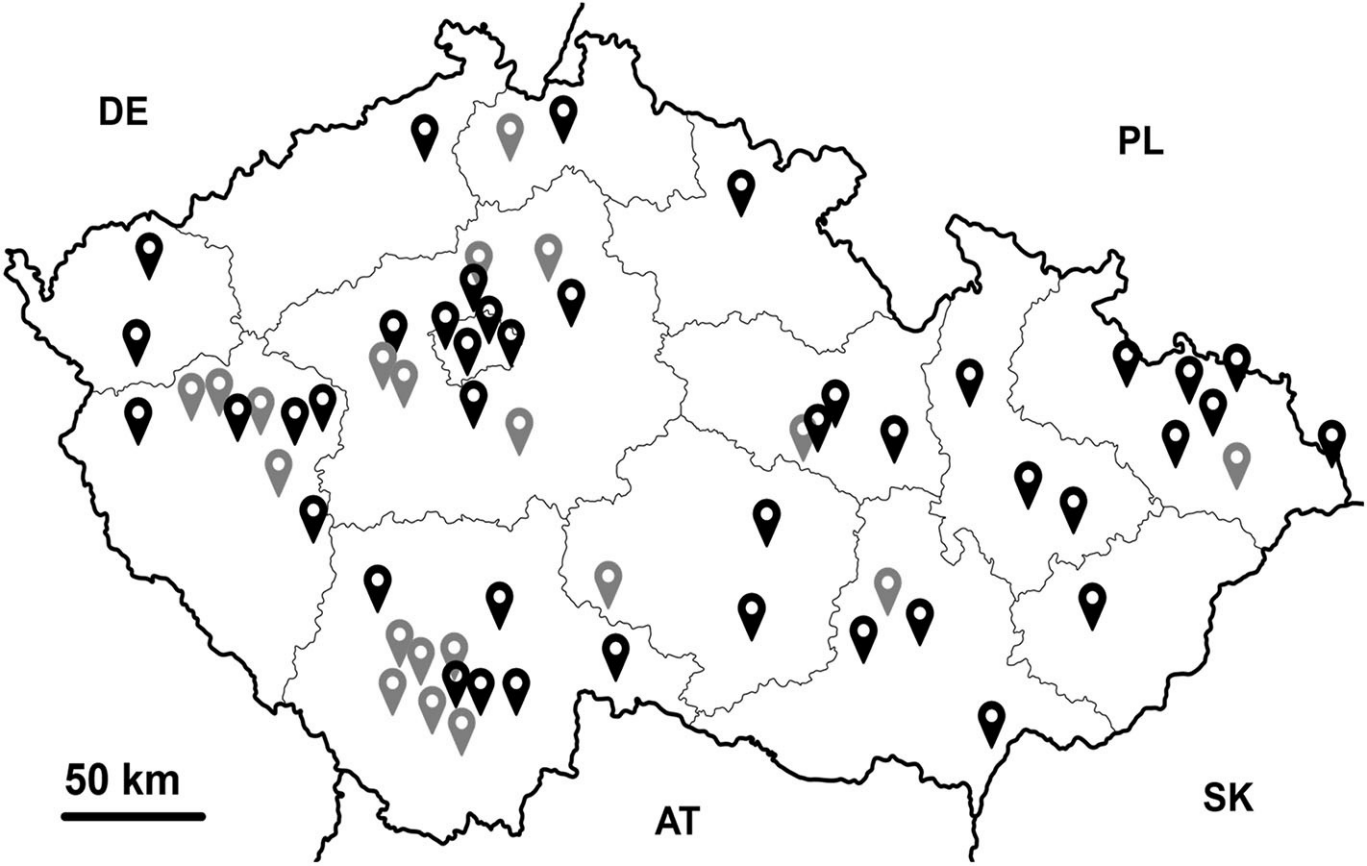
Graphical visualization of regions/localities of the Czech Republic from which samples were obtained. Black indicates samples taken from cities, gray samples from villages.

In total, we obtained 424 samples of which 288 samples were from humans and 136 samples were from non-human hosts (Table 1). Most non-human samples originated from dogs (58/136), followed by cats (19/136) and horses (15/136). Human samples were divided into eight consecutive age categories (Table 2); the majority of samples were from individuals older than 18 years. We obtained 120 samples from men and 168 from women from 14 regions of the Czech Republic (for more details see Figure 1). All volunteers confirmed that they are free of GI symptoms or chronic inflammatory bowel diseases.

**Table 1:** List of human and animal species included in this study and the number of *Blastocystis*-positive samples for each species.

**Host**	**Category**	***N***	**N positive**	**ST1**	**ST2**	**ST3**	**ST4**	**ST5**	**ST6**	**ST7**	**ST8**	**ST10**	**ST14**	Mix^*^	**ST?**
*Homo sapiens*	Asymptomatic	288	70 (24%)	13/70	11/70	25/70	7/70	1/70	5/70	6/70	1/70	–	–	1/70	–
*Felis silvestris catus*	Cats	19	0	–	–	–	–	–	–	–	–	–	–	–	–
*Canis lupus familiaris*	Dogs	58	2 (3%)	–	–	–	–	–	–	2/2	–	–	–	–	–
*Capra aegagrus hircus*	Goats	4	1 (25%)	–	–	–	–	–	–	–	–	1/1	–	–	–
*Anas platyrhynchos domesticus*	Birds	1	0	–	–	–	–	–	–	–	–	–	–	–	–
*Anser anser domesticus*	Birds	1	1 (100%)	–	–	–	–	–	–	1/1	–	–	–	–	–
*Gallus gallus domesticus*	Birds	8	0	–	–	–	–	–	–	–	–	–	–	–	–
*Psittacus erithacus*	Birds	1	0	–	–	–	–	–	–	–	–	–	–	–	–
*Columba livia domestica*	Birds	1	0	–	–	–	–	–	–	–	–	–	–	–	–
*Sus scrofa domestica*	Pigs	3	3 (100%)	–	–	–	–	3/3	–	–	–	–	–	–	–
*Bos primigenius taurus*	Cattle	2	2 (100%)	–	–	–	–	–	–	–	–	1/2	–	1/2	–
*Ovis aries*	Sheep	6	3 (50%)	–	–	–	–	–	–	–	–	1/3	1/3	1/3	–
*Oryctolagus cuniculus domesticus*	Rabbits	13	0	–	–	–	–	–	–	–	–	–	–	–	–
*Phodopus sungorus*	Rodents	1	0	–	–	–	–	–	–	–	–	–	–	–	–
*Cavia aperea porcellus*	Rodents	2	0	–	–	–	–	–	–	–	–	–	–	–	–
*Equus caballus*	Horses	15	1 (7%)	–	–	–	–	–	–	–	–	–	–	–	1/1
*Atelerix albiventris*	Insectivores	1	0	–	–	–	–	–	–	–	–	–	–	–	–

*(N, total number of samples obtained and analyzed within the given category; N positive, number of positive samples for Blastocystis sp. confirmed by sequencing)*.

* *Mix—total number of samples with two or more different subtypes; human ST1 + ST3; cattle ST10 + ST14; sheep ST10 + ST14*.

**Table 2:** Prevalence and incidence of *Blastocystis* sp. subtypes in humans based on the age groups.

**Age**	**Prevalence**	**Subtype**	**Age**	**Prevalence**	**Subtype**
0–3	6% (1/18)	ST3–1/1 (100%)	18–30	19% (14/73)	ST1–3/14 (21%)
					ST2–1/14 (7%)
					ST3–6/14 (43%)
					ST4–3/14 (21%)
					Mix−1/14 (7%)
4–6	45% (5/11)	ST1–1/5 (20%)	31–49	25% (21/83)	ST1–5/21 (24%)
		ST2–1/5 (20%)			ST2–2/21 (10%)
		ST4–1/5 (20%)			ST3–7/21 (33%)
		ST6–1/5 (20%)			ST5–1/21 (5%)
		ST7–1/5 (20%)			ST6–3/21 (14%)
					ST7–2/21 (10%)
					ST8–1/21 (5%)
7–12	31% (5/16)	ST1–2/5 (40%)	50–60	31% (11/36)	ST1–1/11 (9%)
		ST2–1/5 (20%)			ST2–2/11 (18%)
		ST3–1/5 (20%)			ST3–5/11 (46%)
		ST7–1/5 (20%)			ST4–1/11 (9%)
					ST7–2/11 (18%)
13–17	25% (1/4)	ST2–1/1 (100%)	<60	26% (12/47)	ST1–1/12 (8%)
					ST2–3/12 (25%)
					ST3–5/12 (42%)
					ST4–2/12 (17%)
					ST6–1/12 (8%)

*Age categories are divided into eight categories: (i) 0–3, infancy + toddler age; (ii) 4–6, preschool age; (iii) 7–12, younger school age; (iv) 13–17, adolescence; (v) 18–30, young adult age; (vi) 31–49, active age; (vii) 50–60, middle age; (viii) >60, retirement*.

Regarding information about traveling, 52/288 individuals (18%) reported never to have traveled outside the Czech Republic, while 135 (47%) reported having traveled within Europe, and 101 (35%) participants reported that they had traveled outside Europe (Table 3). Eighty-two were village dwellers, while 206 were living in urban environments (Table 3). Furthermore, 244 people reported recent animal contact, of whom 155 had been in contact only with pets and 89 also with farm animals (Table 3). A total of 69 families ranging from 2 to 5 family members were represented in the sample dataset (for details see Supplementary Material 2).

**Table 3:** Prevalence and subtype diversity of *Blastocystis* sp. in human samples according to the specific categories such as lifestyle (village vs. city life, traveling) and contact with animals (pets, farm animals).

**Category**	Sample N^*^	**Prevalence**	**Subtype**	**Category**	Sample N^*^	**Prevalence**	**Subtype**	**Category**	Sample N^*^	**Prevalence**	**Subtype**
**Living locality**						**Non-travelers**					
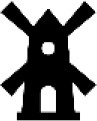 Village	82/288	32% (26/82)	ST1–7/26 (27%)	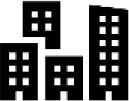 City	206/288	21% (44/206)	ST1–6/44 (14%)	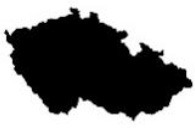 Only in Czech Republic	52/288	21% (11/52)	ST1–3/11 (27%)
			ST2–4/26 (15%)				ST2–7/44 (16%)				ST2–2/11 (18%)
			ST3–7/26 (27%)				ST3–18/44 (41%)				ST3–4/11 (36%)
			ST4–1/26 (4%)				ST4–6/44 (14%)				ST4–1/11 (9%)
			ST5–1/26 (4%)				ST6–2/44 (5%)				ST6–1/11 (9%)
			ST6–3/26 (12%)				ST7–4/44 (9%)				
			ST7–2/26 (8%)				ST8–1/44 (2%)				
			Mix−1/26 (4%)								
**Contact with animals**						**Travelers**					
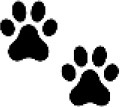 In contact with animals	244/288^**^	25% (62/244)	ST1–13/62 (21%)	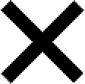 No contact with animals	44 / 288	18% (8/44)	ST2–1/8 (13%)	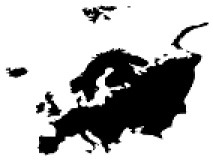 Inside Europe	135 / 288	21% (28/135)	ST1–4/28 (14%)
			ST2–10/62 (16%)				ST3–3/8 (38%)				ST2–3/28 (11%)
			ST3–22/62 (36%)				ST6–1/8 (13%)				ST3–12/28 (43%)
			ST4–7/62 (11%)				ST7–3/8 (38%)				ST4–2/28 (7 %)
			ST5–1/62 (2%)								ST5–1/28 (4%)
			ST6–4/62 (7%)								ST6–2/28 (7%)
			ST7–3/62 (5%)								ST7–4/28 (14%)
			ST8–1/62 (2%)								
					Mix−1/62 (2%)						
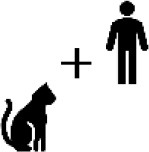 In contact with pets	155/244	21% (33/155)	ST1–4/33 (12%)	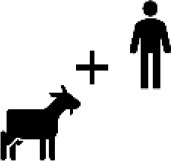 In contact with farm animals	89 / 244^*^	33% (29/89)	ST1–9/29 (31%)	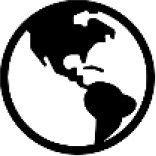 Outside Europe	101/288	31% (31/101)	ST1–6/31 (19%)
			ST2–5/33 (15%)				ST2–5/29 (17%)				ST2–6/31 (19%)
			ST3–16/33 (49%)				ST3–6/29 (21%)				ST3–9/31 (29 %)
			ST4–4/33 (12%)				ST4–3/29 (10%)				ST4–4/31 (13%)
			ST6–1/33 (3%)				ST5–1/29 (3%)				ST6–2/31 (7%)
			ST7–2/33 (6%)				ST6–3/29 (10%)				ST7–2/31 (7%)
			ST8–1/33 (3%)				ST7–1/29 (3%)				ST8–1/31 (3%)
							Mix−1/29 (3%)				Mix−1/31 (3%)

* Sample N, number of analyzed samples in the given category/total number of obtained samples;

** *244, the total number of volunteers which were in contact with animals*.

### Comparison of Sensitivity of Two Diagnostic Methods

Cultivation revealed *Blastocystis* sp. in 73 samples (for more details see Supplementary Material 1). PCR was positive in 100 samples, but sequencing confirmed the presence of *Blastocystis* sp. in 83 samples (Table 1, Supplementary Material 1). Hence, the molecular approach conferred the benefit of higher sensitivity than cultivation, however at slightly lower precisions because it also resulted in 17 false-positive samples (sequences from bacteria, fungi, or plant amplified rather than *Blastocystis* sp.).

### Prevalence and Subtypes of *Blastocystis* sp.

#### Overall Prevalence and Diversity

*Blastocystis* sp. was detected in samples from both human and non-human hosts. The overall prevalence of *Blastocystis* sp. in human samples was 24% (70/288), while 10% (13/136) in non-human hosts, including birds (*Anser anser domesticus*), dogs (*Canis lupus familiaris*), goats (*Capra aegagrus hircus*), pigs (*Sus scrofa domestica*), sheep (*Ovis aries*), cattle (*Bos primigenius taurus*), and horses (*Equus caballus*) (Table 1).

**Figure 2:**
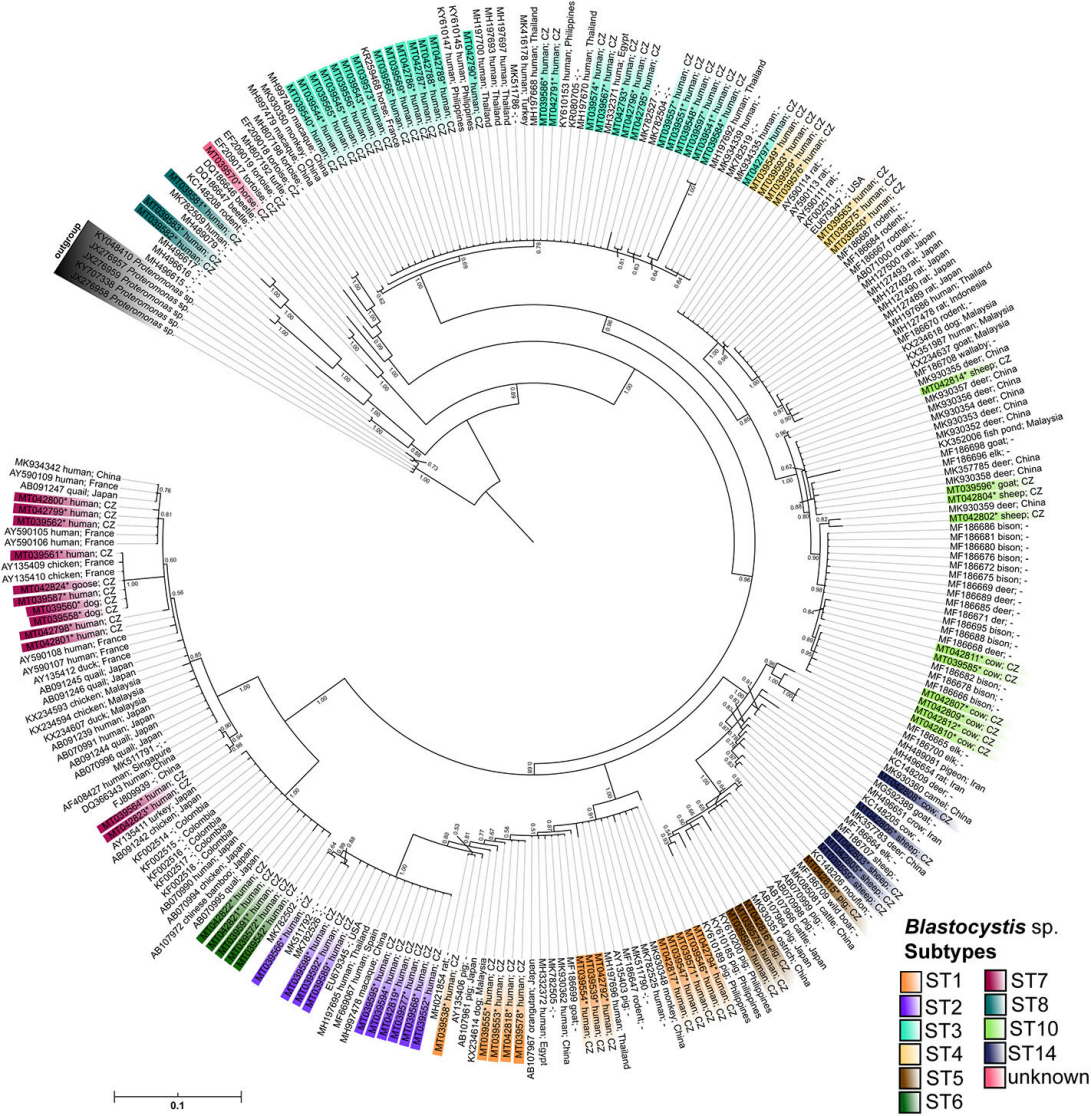
The MrBayes tree based on *Blastocystis* SSU rDNA sequences. The posterior probabilities are shown as a branch supports; sequences of *Proteromonas* sp. were used as an outgroup to root the final tree. Sequences obtained in a frame of this study are marked by the asterisk and highlighted by the color gradient corresponding to the assigned *Blastocystis* subtypes. All sequences are labeled by NCBI accession numbers, the host, and locality if available.

Combining the results obtained by barcoding and phylogenetic analyses, we identified ten different *Blastocystis* sp. subtypes, specifically ST1-ST8, ST10, and ST14 (Tables 1, 3, Figure 2) along with one unidentified subtype in a horse. Most of the results were confirmed using both approaches; however, five sequences were identified to subtypes level by phylogenetic analysis (for details see Supplementary Material 1). While we detected eight subtypes (ST1–ST8) in humans, only five subtypes (ST5, ST7, ST10, ST14, ST?) were revealed in non-human hosts. However, for a few samples, Sanger chromatograms with multiple signals were obtained, which is why the PCR products reflecting these samples were cloned. In three cases, we identified co-colonization by two distinct subtypes of *Blastocystis* sp., specifically ST10 and ST14 in sheep (sequences MT042802–MT042806) and cow (sequences MT042807–MT042812), as well as a mix of ST1 and ST3 in one human sample (sequences MT042792-MT042796; for details see Supplementary Material 1). Moreover, we found one unidentified *Blastocystis* sp. subtype from horse, which in the phylogenetic tree formed a well-separated clade with the sequences obtained from homoiotherms (MH807192, -98, EF209017, -18, -19; Figure 2). Here, the presented phylogenetic results are BI analyzes generated by the software MrBayes (Figure 2) because ML provided very similar results but with somewhat lower branch bootstrap supports (Supplementary Material 3).

The most frequent subtype in humans was ST3 with prevalence of 36% (25/70), followed by ST1 with prevalence 19% (13/70) and ST2 with prevalence 16% (11/70). Interestingly, we detected sizable number of typically “avian subtypes” (ST6 and ST7) in human samples (11/70). Furthermore, we also identified ST5 in humans, which is most commonly found in pigs. Phylogenetic analyses also revealed the presence of subtype ST8 in one human sample (sequences MT039581–MT039583; Figure 1). All these results are displayed in Table 1. The complete alignment of the used sequences is published in the Dryad database (doi: 10.5061/dryad.np5hqbzqv).

#### Detected Alleles of *Blastocystis* sp. Subtypes

We detected a number of different *Blastocystis* subtype alleles in our sample set (Table 4). The most variable subtypes were ST7 (alleles 41, 106, 110, 112) and ST2 (alleles 9, 11, 12), followed by ST4 (42, 92) and ST5 (17, 119). In contrary, the least variable subtypes were ST1 (4) and ST6 (123). In case of ST10, we found the allele 43 using the barcoding platform, but our phylogeny indicates the possible presence of another allele. Within the ST10 clade there are two well-supported branches, one of which has a posterior probability (PP) value 0.85 containing only one sample (MT042802; unidentified allele), while other sequences reflecting allele 43 fall into the second branch (*PP*-value 0.98; Figure 2). The ST14 subtype (MT042803, -05, -06, -08, MT039559) was revealed only using phylogenetic analysis, and also here, the high-supported branches with the clade could indicate the presence of at least two alleles (Figure 2).

**Table 4:** Intra-subtype variability of detected *Blastocystis* subtypes according to host.

**ST**	**Host**	**N**	**Alleles**	**ST**	**Host**	**N**	**Alleles**
ST1	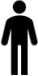	13	13× allele 4	ST7	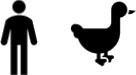	9	2× allele 41 (ho)
							5× allele 112 (2× ho, 2× ca, 1× an)
							1× allele 110 (ho)
							1× allele 106 (ho)
ST2	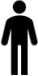	11	5× allele 9	ST8	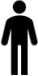	1	1× allele X
			5× allele 11				
			1× allele 12				
ST3	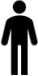	24	23× allele 34	ST10	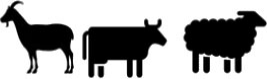	3	2× allele 43 (ov, bo)
			1× allele 38				1x allele X (cp)
ST4	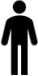	7	6× allele 42	ST14	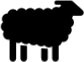	1	1× allele X (ov)
			1× allele 92				
ST5	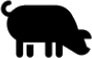	2	1× allele 17	Mix	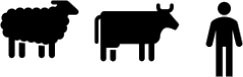	5	ST10 (allele X) + ST14 (allele X) (ov)
			1× allele 119				ST10 (43) + ST14 (X) (bo)
							ST5 (119) + ST5 (17) (ho)
							ST1 (4) + ST3 (34) (ho)
							ST3 (36) + ST3 (34) (ho)
ST6	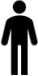	5	4× allele 123	ST?	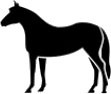	1	1x allele X
			1× allele X				

*(ST, subtype; N, total number of positive samples in the given category; ho, human; ca, dog; an, goose; ov, sheep; bo, cattle; cp, goat)*.

Surprisingly, we found two different alleles within one subtype in two human samples - ST5 with alleles 119 and 17 in the first case, and ST3 with alleles 36 and 34 in the second case, which is a very rare finding.

#### Distribution of *Blastocystis* sp. Within Family Members

We were able to obtain a relatively large number of samples from more family members, and it was therefore possible to determine whether *Blastocystis* sp. is likely to circulate within families. In total, we obtained samples from 69 families (for details see Supplementary Material 2). At least one *Blastocystis*-positive person was observed in approximately half of the families (35/69). In most cases, only one family member was positive for *Blastocystis* sp. (25/35), but there were also families with more positive members (10/35). In the latter group of families, more subtypes occurred in several family members at the same time (6/35). For example, three different subtypes were detected in a family of four [ST1 (4), ST2 (9), ST6 (123)], and a similar situation occurred in another family consisting of five members [ST1 (4), ST2 (11), 2× ST4 (42)]. Identical subtypes of multiple family members were found in four cases (4/69) only. One family was living in a city where the married couple shared ST3 with the same allele 34. Further two families living in the village–both families of three - all had the ST1 exhibiting the same allele 4. The last case was the mother and her daughter from the small village shearing ST2, allele 9.

### Influence of Specific Factors on the Occurrence of *Blastocystis* sp.

#### Lifestyle

All 288 human samples were tested for the effect of lifestyle on the presence/absence of *Blastocystis* sp. Specifically, two factors were included in this category: (i) town/city life versus village life and (ii) the impact of traveling. A significantly higher prevalence (*p* = 0.03) was found among people living in village areas (32%), compared to those from living in urban areas (21%). The most common subtypes in individuals from village areas were ST1 and ST3 (both 27%) and ST2 (15%); in urban areas, ST3 (41%), ST2 (16%), and ST1 and ST4 (14%) (Table 3). Compared to people living in a town/city, we detected a higher number of ST6 cases in village people (12 vs. >5%); ST6 is considered one of the two subtypes that are considered “avian,” the other one being ST7 (Stensvold et al., 2009).

Regarding the impact of traveling on differences in *Blastocystis* sp. colonization, the highest prevalence (31%; *p* = 0.11) was observed among the group of people traveling outside the Europe (Table 3). The prevailing subtype in this category was ST3 (29%), followed by ST1 and ST2 (both 19%). There was no difference in the prevalence of *Blastocystis* sp. (*p* = 0.48) between groups that do not travel or travel only within the Europe only (21% in both categories; Table 3). In both latter groups, the most common subtype was also ST3 (36 and 43%, respectively). The second most common subtype in people traveling within the Europe was ST1 and ST7 (both 14%; Table 3).

#### Contact With Animals

The prevalence of *Blastocystis* sp. among people in close contact with animals was 25%, while 18% (*p* = 0.15) for those without contact with animals (Table 3): the most common subtypes in the first group (contact with animals) were ST3 (36%), ST1 (21%), and ST2 (16%); in the second group (without contact with animals) ST3 and ST7 (both 38%), then ST2 and ST6 (both 13%; Table 3). The group of people in contact with animals was further divided into two subgroups – contact with (i) pets and (ii) farm animals. A significantly higher prevalence was observed in humans in contact with farm animals (33%), compared to people in contact with pets (21%, *p* = 0.0256; Table 3). In people with pets, the most common subtype is ST3 (49%), followed by ST2 (15%), then ST1 and ST4 (both 12%). In the second group (contact with farm animals), the most frequent were ST1 (31%), ST3 (21%), and ST2 (17%; Table 3). For samples from animals belonging to individuals / whole families with cases that were *Blastocystis*-positive, no subtypes were found to be shared between the human and non-human hosts (for details see Table 4).

#### Age and Gender

The highest prevalence of *Blastocystis* sp. was observed in the age group of 4–6 years (45%, *p* = 0.43), although this result was not statistically significant (Table 2). Another age category with a relatively high prevalence of this protist is among subject aged 7–12 and 50–60 years (31%; Table 2). In contrast, the least positive cases were found in children under three years (6%). Complete results of the prevalence and incidence of *Blastocystis* sp. subtypes in each age group are summarized in Table 2.

Regarding influence of gender, there was no significant difference (*p* = 0.12) in the prevalence of *Blastocystis* sp. between male (21%) and female (27%). The most common subtype was ST3 in both groups (40% and 33%), then ST1 (28%) and ST2 (12%) in males, ST2 (18%), and ST1 (13%) in females.

## Discussion

Over the past decade, there has been a growing interest in investigating the diversity of commensal unicellular eukaryotes in the healthy human population partly due to the hypothesis that one or more of these genera/species could be conducive to human health (Lukeš et al., 2015; Chabé et al., 2017; Stensvold and van der Giezen, 2018). Of the wide range of these gut micro-eukaryotic genera, *Blastocystis* sp. is the one most intensively studied (e.g., Stensvold and van der Giezen, 2018), and recently, it has been associated with the diversity of bacterial microbiota (e.g., Andersen et al., 2015, 2016; Audebert et al., 2016; Chabé et al., 2017). In order to fill gaps in our knowledge on the role of commensal intestinal protists in the gut ecosystem, we need to explore extensively their prevalence in healthy individuals across the globe, in countries with different stages of industrialization. Therefore, the aim of the present study was to expand the knowledge of the epidemiology of *Blastocystis* sp. in gut-healthy asymptomatic humans across the Czech Republic (belonging among the industrialized countries), including the subtype distribution of *Blastocystis* sp., and correlations of *Blastocystis* sp. colonization with several factors such as lifestyle (traveling, city versus village life), contact with animals, as well as age and gender.

In this study, a total of 288 stool samples from asymptomatic individuals (gut healthy) were obtained across the entire age range (between several months of age to more than 60 years) and 136 samples from animals with which volunteers were in contact on a daily basis. For the detection and differentiation of *Blastocystis* sp., we chose two methodological approaches, specifically xenic cultivation and molecular detection (PCR and sequencing). PCR identified about ten more positive samples than cultivation. This fact corresponds with other studies (e.g., Stensvold et al., 2007; Roberts et al., 2013), where molecular diagnostic is more sensitive than cultivation.

The overall prevalence of *Blastocystis* sp. in our human sample set reached 24% which corresponds well with prevalence data from studies performed in other European countries ranging between 18 and 30% (Bart et al., 2013; El Safadi et al., 2016; Tito et al., 2019) but significantly differs from a recent study in the US with prevalence 7% (Scanlan et al., 2016). Surprisingly, a much higher prevalence was found in an Irish population of healthy people, 56% (Scanlan and Marchesi, 2008; Scanlan et al., 2014). In contrast, *Blastocystis* sp. occurred less frequently in animals in our sample-set (10%) compared to other studies where prevalence ranged from 18–76% (Roberts et al., 2013; Udonsom et al., 2018; Valença-Barbosa et al., 2019). This difference could be explained by the fact that a large proportion of our animal samples were from cats and dogs, in which *Blastocystis* sp. is generally a much less common finding (about 3%) than it is in livestock (ranging between 12 and 76 %) (Wang et al., 2013; Paulos et al., 2018; Udonsom et al., 2018; Greige et al., 2019). Unfortunately, due to limited communication in most volunteers, we were not able to monitor *Blastocystis*-positive individuals for a longer period to determine, for example, the length of *Blastocystis* sp. colonization (i.e., whether it is time-limited or not) or to determine if they have occasional symptomatic periods.

Overall, ten different *Blastocystis* subtypes (ST1–ST9 and ST12) have been found in humans (Alfellani et al., 2013a; Stensvold et al., 2020a), of which we detected eight in this study, specifically ST1–ST8. Our results on the subtype distribution across human individuals are in an agreement with findings from similar studies (e.g., Bart et al., 2013; El Safadi et al., 2016). However, in humans, more frequently is detected a lower diversity of subtypes, mostly ST1-ST3 (Scanlan et al., 2016; Jalallou et al., 2017; Mohammad et al., 2017) or ST1-ST4 (Scanlan et al., 2014; Valença Barbosa et al., 2017). Comparing data on occurrence and distribution *Blastocystis* sp. between various studies is often very difficult, maybe almost impossible, because of inconsistency in structure of human cohorts [often monitored mainly individuals with intestinal disorders; e.g., Krogsgaard et al. (2015)], in countries of origin [developmental or industrialized; e.g., Scanlan et al. (2014)], in geographic localizations (rural or urban; Alfellani et al., 2013b) or in use of wide range of diagnostic approaches (Stensvold and Clark, 2016b).

Interestingly, this study allowed us to see if *Blastocystis* sp. and its subtypes could circulate within family members. In total, we obtained samples from 69 families and detected at least one *Blastocystis* sp. positive individual in 35, of which ten showed more colonized individuals. Interestingly, in about six families we also discovered more *Blastocystis* subtypes within one family. These results might suggest that *Blastocystis* sp. circulates between family members or all family members could have been colonized from the same source.

In the present study, the most common subtype in the human sample set was ST3 (36%), followed by ST1 (19%) and ST2 (16%). This result corresponds with data from other European studies where subtypes ST1-ST4 were the most common (Forsell et al., 2012; Bart et al., 2013; Scanlan et al., 2014; El Safadi et al., 2016). These four common subtypes in humans have been also found in other hosts such as primates, ungulates, rodents or birds, which could indicate zoonotic potential (Stensvold and Clark, 2016a). However, for subtypes ST1, 3, and 4, allele analysis to a large extent indicated that the strains detected here were the typical ones circulating between humans. Nevertheless, our finding of the avian subtypes ST6 and ST7 in humans as well as ST5 (often found in pigs) and ST8, could reflect cases of zoonotic transmission (Stensvold and Clark, 2016a). Interestingly, rats might play a role in spreading of *Blastocystis* ST1-ST4 and possible also of ST8 as suggested by a study focused on a survey of wastewater samples in Sweden (Stensvold et al., 2020b).

The avian subtypes ST6 and ST7 were detected in 16% of human samples (7 and 9%, respectively). These subtypes hardly appear in other studies of humans in Europe (e.g., Forsell et al., 2012; Bart et al., 2013; El Safadi et al., 2016; Paulos et al., 2018). Human colonization with ST6 may occur due to direct contact with birds, for example poultry, which has recently been confirmed to have a relatively high prevalence of ST6 (32%) (Stensvold and Clark, 2016b; Greige et al., 2018; Wang et al., 2018). Moreover, recent evidence has demonstrated the transmission of this subtype between poultry and their keepers (Greige et al., 2018). Here, three out of five ST6-positive people live in a village area, two of which also keep poultry besides other animals. Unfortunately, poultry samples were not available from these volunteers and, thus, it was not possible confirmed the presence of ST6 in these birds. In contrast, another “avian subtype” ST7 was detected in six people living mainly in towns/cities (4/6). Only three of them reported contact with animals, specifically dogs/cats (3/6), poultry and livestock (1/6). In this case, we obtained only one fecal sample from the dog and one from the cat, however, both appeared to be *Blastocystis*-negative. Beside this, we recorded ST7 in other two dogs and one goose, but conversely to previous case the presence of this subtype was not confirmed in their owners. So far, most of the epidemiological studies focused on the occurrence of *Blastocystis* sp. in dogs found either different subtypes, such as ST1, ST2, and ST4 (Wang et al., 2013, 2018; Ramírez et al., 2014), or the presence of *Blastocystis* sp. was not confirmed at all (Paulos et al., 2018). Despite of all above discussed facts, it is important to mention that ST6 and ST7 were also detected in other animal species (bovids, carnivores, or non-human primates) which might be also reservoirs of *Blastocystis* sp. (e.g., Cian et al., 2017; Wang et al., 2018).

Other unexpected results were findings ST5 and ST8 always in only one human sample while the presence of ST8 was confirmed only by phylogenetic analyses. Typical host for subtype ST5 is livestock, but mainly pigs (Noël and Dufernez, 2005; Yan et al., 2007; Wang et al., 2014; Lee et al., 2018; Udonsom et al., 2018; Greige et al., 2019). The individual positive for ST5 in this study lived in a village and owned a small family farm, so, we hypothesize about zoonotic transmission. Moreover, we found ST5 with same alleles (17, 119) also in two pigs. In case of ST8, typical subtype of zookeepers of non-human primates (Stensvold and Clark, 2016b), we revealed this colonization only in one woman. She has no history of contact with primates, however, often travels outside the Europe.

The *Blastocystis* sp. subtypes found in this study were delineated to 17 different alleles. However, eight sequences showed no match in the barcoding platform, therefore, those were subjected to phylogenetic. The highest intra-subtype variability in our sample-set was in ST7 (alleles 41, 106, 110, 112), followed by ST2 (alleles 9, 11, 12). While larger diversity of alleles within ST2 isolates (in contrast to commonly occurring ST1 and ST4) was revealed also in some other studies (Ramírez et al., 2017; Rezaei Riabi et al., 2018), such high intra-subtype diversity of ST7 is not so common (e.g., Ramírez et al., 2017; Rezaei Riabi et al., 2018). Interestingly, we detected one allele (112) of subtype ST7 in both humans and animals (specifically in two dogs and one goose) which might indicate a possible zoonotic transmission.

It is generally assumed that people living in rural areas are more likely to encounter potential sources of *Blastocystis* sp. such as contaminated water and food probably due to closer contact with animals (e.g., Parkar et al., 2010; Angelici et al., 2018; Greige et al., 2018, 2019). The results of our study demonstrate a significantly higher prevalence in people living in a village (32%), compared to those from a town/city where the prevalence of *Blastocystis* sp. was only 21%. Similar results were observed in Brazil, with a prevalence of 35% detected in Rio de Janeiro (Valença Barbosa et al., 2017), while in two small fishing villages in São Paulo the prevalence was higher (45 and 71%) (David et al., 2015). An interesting finding in our dataset is a 7% higher incidence of subtype ST6 in people from the village, compared to people who reported town/city life in the questionnaire. A similar result was found in a Turkish study comparing the diversity of *Blastocystis* sp. subtypes depending on lifestyle (rural/urban) (Koltas and Eroglu, 2016). The authors of this study detected ST6 together with the other two subtypes (ST5 and ST7) only in rural people. These facts may support our hypothesis that poultry is the main source of ST6 for humans (Greige et al., 2018), but this requires further investigation.

In relation to lifestyle factor, there was observed correlations between the presence/absence of *Blastocystis* sp. and traveling mainly. Generally speaking, it is believed that traveling in tropical and subtropical countries (mainly Africa and Asia) may influence the occurrence of the *Blastocystis* sp. in humans (El Safadi et al., 2016; Rudzińska et al., 2019). This fact was also confirmed in our study. The highest prevalence (31%) of *Blastocystis* sp. in our sample-set was observed among the group of people traveling outside the Europe that correlates with other studies (Bart et al., 2013; Sekar and Shanthi, 2015; El Safadi et al., 2016). In contrast, there was no significant difference in the prevalence of *Blastocystis* sp. among the group of people that do not travel or travel only within the Europe. This result is probably related to the fact that the prevalence of *Blastocystis* sp. in Europe and, thus, in the Czech Republic (based on our data) is similar. Some studies report a significant higher occurrence of ST1 in individuals traveling outside of Europe (Bart et al., 2013; Rudzińska et al., 2019), however, we found no such correlation. Traveling outside the Europe is also often correlated with the occurrence of “avian” subtypes ST6 and ST7 (e.g., Rudzińska et al., 2019; van Hattem et al., 2019), but we did not confirm this.

There is no consensus on the impact of gender on the occurrence of *Blastocystis* sp. in humans. While some studies report significant differences in the prevalence of *Blastocystis* sp. between men and women (e.g., Abdulsalam et al., 2013; Rudzińska et al., 2019), others do not confirm such a difference (e.g., Scanlan et al., 2014; El Safadi et al., 2016; Paulos et al., 2018). In our study, there was no significant difference in the occurrence of *Blastocystis* sp. between the sexes, although a slightly higher prevalence was found in women (27%) than in men (21%).

In case of age factor, the highest prevalence (45%) of *Blastocystis* sp. among our human samples was in the category 4-6 years. Nevertheless, this can be caused by the smaller number of samples in this group and no major conclusions can be drawn. The authors of the French study also found a higher prevalence (27%) in children under 14 years (El Safadi et al., 2016). Authors of the study Paulos et al. (2018) had a similar experience with the highest prevalence in 6–10 and 11–15-years old children (55 and 52%). The second highest prevalence of *Blastocystis* sp. in our samples was detected in the 50–60 age group with a value of 35%, which differs from the results of other studies–for example, in the above-mentioned study from France, these protists were only in 13.6% of people over 50.

In contrast, in children under three years (in our study), *Blastocystis* sp. was the least prevalent and only one sample (out of 18) was positive. This result is consistent with the data published in a recent study from Ireland (Scanlan et al., 2018) in which none of the infants (>1 year) were positive and these authors also found low prevalence in children under two years (only 5%). Zero prevalence in infants was also confirmed by a study conducted in India (Pandey et al., 2015). Such young children are under the constant supervision of their parents and usually do not have as many opportunities to encounter infection as older children, which may be the reason for low prevalence in this age group.

### Conclusion

In sum, the results of our study on the prevalence and distribution of *Blastocystis* sp. and its subtypes in a gut-healthy human population in the Czech Republic more or less correlate with data in other epidemiological studies from industrialized countries. However, a comparison of the results from barcoding with phylogenetic analysis emphasis the need to focus further research on possible sources of *Blastocystis* sp. for human colonization, and in particular on the possibility of zoonotic transmissions. An in-depth insight to these aspects will significantly contribute to understanding the importance of *Blastocystis* sp. in human health, its role in the intestinal microbiome and its epidemiology across urban a rural localities.

## Data Availability Statement

The datasets presented in this study can be found in online repositories. The names of the repository/repositories and accession number(s) can be found in the article/Supplementary Material.

## Ethics Statement

The studies involving human participants were reviewed and approved by Ethics Committee of the Biology Center of the Czech Academy of Sciences (reference number: 1/2017). Written informed consent to participate in this study was provided by the participants' legal guardian/next of kin.

## Author Contributions

ZL, OH, and KB: field work. ZL, MJ, KJ, and CS: conceptualization. ZL, OH, KB, MJ, and MK: methodology and investigation. ZL, KJ, and MK: funding acquistion and project administration. ZL, MK, and CS: software. ZL, MJ, MK, CS, and KJ: validation. ZL, MJ, CS, and KJ: draft writing. All authors contributed to the article and approved the submitted version.

## Conflict of Interest

The authors declare that the research was conducted in the absence of any commercial or financial relationships that could be construed as a potential conflict of interest.

## Supplementary Material

Supplementary Material 1 Comparison of barcoding and phylogenetic results on *Blastocystis* subtypes.

Supplementary Material 2 Distribution of *Blastocystis* sp. within family members.

Supplementary Material 3 The ML tree based on *Blastocystis* SSU rDNA sequences, statistical support for each branch was obtained from maximum-likelihood bootstrap; supports lower than 200 are not shown. Sequences of *Proteromonas* sp. were used as an outgroup to root the presented tree; data obtained for this study are highlighted by bold.

## References

[B1] Abdulsalam A. M. Ithoi I. Al-Mekhlafi H. M. Khan A. H. Ahmed A. Surin J.. (2013). Prevalence, predictors and clinical significance of *Blastocystis* sp. in sebha, libya. Parasit. Vectors 8:86. 10.1186/1756-3305-6-86 23566585 PMC3626707

[B2] Alexeieff A. (1911). Sur nature des formations dites kystes de *Trichomonas intestinalis*. C. R. Soc. Biol. 71, 296–298.

[B3] Alfellani M. A. Stensvold C. R. Vidal-Lapiedra A. Onuoha E. S. U. Fagbenro-Beyioku A. F. Clark C. G. (2013b). Variable geographic distribution of *Blastocystis* subtypes and its potential implications. Acta Trop. 126, 11–18. 10.1016/j.actatropica.2012.12.011 23290980

[B4] Alfellani M. A. Taner-Mulla D. Jacob A. S. Imeede C. A. Yoshikawa H. Stensvold C. R.. (2013a). Genetic diversity of *Blastocystis* in livestock and zoo animals. Protist 164, 497–509. 10.1016/j.protis.2013.05.003 23770574

[B5] Andersen L. O. Bonde I. Nielsen H. B. Stensvold C. R. (2015). A retrospective metagenomics approach to studying *Blastocystis*. FEMS Microbiol. Ecol. 91:fiv072. 10.1093/femsec/fiv072 26130823

[B6] Andersen L. O. Karim A. B. Roager H. M. Vigsnaes L. K. Krogfelt K. A. Licht T.R.. (2016). Associations between common intestinal parasites and bacteria in humans as revealed by qPCR. Eur. J. Clin. Microbiol. Infect. Dis. 35, 1427–1431. 10.1007/s10096-016-2680-2 27230509

[B7] Angelici M. C. Nardis C. Scarpelli R. Ade P. (2018). *Blastocystis hominis* transmission by non-potable water: a case report in Italy. New Microbiol. 41, 173–177. 29498738

[B8] Audebert C. Even G. Cian A. Loywick A. Merlin S. Viscogliosi E.. (2016). Colonization with the enteric protozoa *Blastocystis* is associated with increased diversity of human gut bacterial microbiota. Sci. Rep. 6:25255. 10.1038/srep25255 27147260 PMC4857090

[B9] Bart A. Wentink-Bonnema E. M. Gilis H. Verhaar N. Wassenaar C. J. van Vugt M.. (2013). Diagnosis and subtype analysis of *Blastocystis* sp. in 442 patients in a hospital setting in the Netherlands. BMC Infect. Dis. 13:389. 10.1186/1471-2334-13-389 23972160 PMC3765316

[B10] Chabé M. Lokmer A. Ségurel L. (2017). Gut protozoa: friends or foes of the human gut microbiota? Trends Parasitol. 33, 925–934. 10.1016/j.pt.2017.08.005 28870496

[B11] Cian A. El Safadi D. Osman M. Moriniere R. Gantois N. Benamrouz-Vanneste S.. (2017). Molecular epidemiology of *Blastocystis sp*. in various animal groups from two French zoos and evaluation of potential zoonotic risk. PLoS ONE 12:e0169659. 10.1371/journal.pone.0169659 28060901 PMC5217969

[B12] Clark C. G. Stensvold C. R. (2016). *Blastocystis*: isolation, xenic cultivation, and cryopreservation. Curr. Protoc. Microbiol. 43, 369–377. 10.1002/cpmc.18 27858970

[B13] Clark C. G. van der Giezen M. Alfellani M. A. Stensvold C. R. (2013). Recent developments in *Blastocystis* research. Adv. Parasitol. 82, 1–32. 10.1016/B978-0-12-407706-5.00001-0 23548084

[B14] David É. B. Guimarães S. De Oliveira A. P. Bittencourt G. N. Nardi A. R. M. Ribolla P. E. M.. (2015). Molecular characterization of intestinal protozoa in two poor communities in the State of São Paulo, Brazil. Parasit. Vectors 8:103. 10.1186/s13071-015-0714-8 25889093 PMC4335703

[B15] El Safadi D. Cian A. Nourrisson C. Pereira B. Morelle C. Bastien P.. (2016). Prevalence, risk factors for infection and subtype distribution of the intestinal parasite *Blastocystis* sp. *from a large-scale multi-center study in France*. BMC Infect. Dis. 16:451. 10.1186/s12879-016-1776-8 27566417 PMC5002209

[B16] El Safadi D. Gaayeb L. Meloni D. Cian A. Poirier P. Wawrzyniak I.. (2014). Children of senegal river basin show the highest prevalence of *Blastocystis* sp. ever observed worldwide. BMC Infect. Dis. 14, 1471–2334. 10.1186/1471-2334-14-164 24666632 PMC3987649

[B17] Forsell J. Granlund M. Stensvold C. R. Clark G. C. Evengard B. (2012). Subtype analysis of *Blastocystis* isolates in Swedish patients. Eur. J. Clin. Microbiol. Infect. Dis. 31, 1689–1696. 10.1007/s10096-011-1416-6 22350386

[B18] Greige S. El Safadi D. Khaled S. Gantois N. Baydoun M. Chemaly M.. (2019). First report on the prevalence and subtype distribution of *Blastocystis* sp. in dairy cattle in lebanon and assessment of zoonotic transmission. Acta Trop. 194, 23–29. 10.1016/j.actatropica.2019.02.013 30878470

[B19] Greige S. Safadi D. El Bécu N. Gantois N. Pereira B. Chabé M.. (2018). Prevalence and subtype distribution of *Blastocystis* sp. isolates from poultry in lebanon and evidence of zoonotic potential. Parasit. Vectors 11:389. 10.1186/s13071-018-2975-5 29973261 PMC6030734

[B20] Guindon S. Gascuel O. (2003). A simple, fast, and accurate algorithm to estimate large phylogenies by maximum likelihood. Syst. Biol. 52, 696–704. 10.1080/10635150390235520 14530136

[B21] Jalallou N. Iravani S. Rezaeian M. Alinaghizade A. Mirjalali H. (2017). Subtypes distribution and frequency of *Blastocystis* sp. isolated from diarrheic and non-diarrheic patients. Iran. J. Parasitol. 12, 63–68. 28761462 PMC5522700

[B22] Jeremiah S. Parija S. (2013). *Blastocystis*: taxonomy, biology and virulence. Trop. Parasitol. 3, 17–25. 10.4103/2229-5070.113894 23961437 PMC3745665

[B23] Jiménez P. A. Jaimes J. E. Ramírez J. D. (2019). A summary of *Blastocystis* subtypes in North and South America. Parasit. Vectors 12:376. 10.1186/s13071-019-3641-2 31358042 PMC6664531

[B24] Katoh K. Kuma K. I. Toh H. Miyata T. (2005). MAFFT version 5: improvement in accuracy of multiple sequence alignment. Nucleic Acids Res. 33, 511–518. 10.1093/nar/gki198 15661851 PMC548345

[B25] Koltas I. S. Eroglu F. (2016). Subtype analysis of *Blastocystis* isolates using SSU rRNA-DNA sequencing in rural and urban population in southern Turkey. Exp. Parasitol. 170, 247–251. 10.1016/j.exppara.2016.10.006 27725159

[B26] Krogsgaard L. R. Andersen L. O'Brien Johannesen T. B. Engsbro A. L. Stensvold C. R.. (2018). Characteristics of the bacterial microbiome in association with common intestinal parasites in irritable bowel syndrome. Clin. Transl. Gastroenterol. 9:161. 10.1038/s41424-018-0027-2 29915224 PMC6006308

[B27] Krogsgaard L. R. Engsbro A. L. Stensvold C. R. Nielsen H. V. Bytzer P. (2015). The prevalence of intestinal parasites is not greater among individuals with irritable bowel syndrome: a population-based case-control study. Clin. Gastroenterol. Hepatol. 13, 507–513. 10.1016/j.cgh.2014.07.065 25229421

[B28] Lee H. Lee S. H. Seo M. G. Kim H. Y. Kim J. W. Lee Y. R.. (2018). Occurrence and genetic diversity of *Blastocystis* in Korean cattle. Vet. Parasitol. 258, 70–73. 10.1016/j.vetpar.2018.06.010 30105981

[B29] Leelayoova S. Siripattanapipong S. Thathaisong U. Naaglor T. Taamasri P. Piyaraj P.. (2018). Drinking water: a possible source of *Blastocystis* spp. subtype 1 infection in schoolchildren of a rural community in central Thailand. Am. J. Trop. Med. Hyg. 79, 401–406. 10.4269/ajtmh.2008.79.401 18784233

[B30] Leelayoova S. Taamasri P. Rangsin R. Naaglor T. Thathaisong U. Mungthin M. (2002). *In-vitro* cultivation: a sensitive method for detecting *Blastocystis hominis*. Ann. Trop. Med. Parasitol. 96, 803–807. 10.1179/000349802125002275 12625935

[B31] Lukeš J. Stensvold C. R. Jirku-Pomajbíková K. Wegener Parfrey L. (2015). Are human intestinal eukaryotes beneficial or commensals? PLoS Pathog. 11:e1005039. 10.1371/journal.ppat.1005039 26270819 PMC4536199

[B32] Maloney J. G. Lombard J. E. Urie N. J. Shivley C. B. Santin M. (2019). Zoonotic and genetically diverse subtypes of *Blastocystis* in US pre-weaned dairy heifer calves. Parasitol. Res. 118, 575–582. 10.1007/s00436-018-6149-3 30483890

[B33] Mardani Kataki M. Tavalla M. Beiromvand M. (2019). Higher prevalence of *Blastocystis hominis* in healthy individuals than patients with gastrointestinal symptoms from Ahvaz, Southwestern Iran. Comp. Immunol. Microbiol. Infect. Dis. 65, 160–164. 10.1016/j.cimid.2019.05.018 31300108

[B34] Mohammad N. A. Al-Mekhlafi H. M. Moktar N. Anuar T. S. (2017). Prevalence and risk factors of *Blastocystis* infection among underprivileged communities in rural Malaysia. Asian Pac. J. Trop. Med. 10, 491–497. 10.1016/j.apjtm.2017.05.001 28647187

[B35] Nieves-Ramirez M. E. Partida-Rodriguez O. Laforest-Lapointe L. A. Reynolds L. A. Brown E. M. Morien E.. (2018). Asymptomatic intestinal colonization with protist *Blastocystis*. mSystems 3, e00007–18. 10.1128/mSystems.00007-18 29963639 PMC6020473

[B36] Noël C. Dufernez F. (2005). Molecular phylogenies of *Blastocystis* isolates from different hosts: implications for genetic diversity, identification of species, and zoonosis. J. Clin. Microbiol. 43, 348–355. 10.1128/JCM.43.1.348-355.2005 15634993 PMC540115

[B37] Nourrisson C. Scanzi J. Pereira B. Nkoudmongo C. Wawrzyniak I. Cian A.. (2014). *Blastocystis* is associated with decrease of fecal microbiota protective bacteria: comparative analysis between patients with irritable bowel syndrome and control subjects. PLoS ONE 9:e111868. 10.1371/journal.pone.0111868 25365580 PMC4218853

[B38] Oliveira-Arbex A. P. David É. B. Guimarães S. (2018). *Blastocystis* genetic diversity among children of low-income daycare center in Southeastern Brazil. Infect. Genet. Evol. 57, 59–63. 10.1016/j.meegid.2017.11.005 29126996

[B39] Pandey P. K. Verma P. Marathe N. Shetty S. Bavdekar A. Patole M. S.. (2015). Prevalence and subtype analysis of *Blastocystis* in healthy Indian individuals. Infect. Genet. Evol. 31, 296–299. 10.1016/j.meegid.2015.02.012 25701123

[B40] Parfrey L. W. Walters W. A. Lauber C. L. Clemente J. C. Berg-Lyons D. Teiling C.. (2014). Communities of microbial eukaryotes in the mammalian gut within the context of environmental eukaryotic diversity. Front. Microbiol. 5:298. 10.3389/fmicb.2014.00298 24995004 PMC4063188

[B41] Parkar U. Traub R. J. Vitali S. Elliot A. Levecke B. Robertson I.. (2010). Molecular characterization of *Blastocystis* isolates from zoo animals and their animal-keepers. Vet. Parasitol. 169, 8–17. 10.1016/j.vetpar.2009.12.032 20089360

[B42] Paulos S. Koster P. C. de Lucio A. Hernandez-de-Mingo M. Cardona G. A. Fernandez-Crespo J. C.. (2018). Occurrence and subtype distribution of *Blastocystis* sp. in humans, dogs and cats sharing household in northern Spain and assessment of zoonotic transmission risk. Zoonoses Public Health 65, 993–1002. 10.1111/zph.12522 30198123

[B43] Petersen A. M. Stensvold C. R. Mirsepasi H. Engberg J. Friis-Møller A. Porsbo L. J.. (2013). Active ulcerative colitis associated with low prevalence of *Blastocystis* and *Dientamoeba fragilis* infection. Scand. J. Gastroenterol. 48, 638–639. 10.3109/00365521.2013.780094 23528075

[B44] Poirier P. Wawrzyniak I. Vivarès C. P. Delbac F. El Alaoui H. (2012). New insights into *Blastocystis* spp. a potential link with irritable bowel syndrome. PLoS Pathog. 8:e1002545. 10.1371/journal.ppat.1002545 22438803 PMC3305450

[B45] Poulsen C. S. Efunshile A. M. Nelson J. A. Stensvold C. R. (2016). Epidemiological aspects of *Blastocystis* colonization in children in Ilero, Nigeria. Am. J. Trop. Med. Hyg. 95, 175–179. 10.4269/ajtmh.16-0074 27139454 PMC4944538

[B46] Rambaut A. (2016). FigTree v1.4.3. Available online at: *http://tree.bio.ed.ac.uk/software/figtree/* *(accessed* April 10, 2016).

[B47] Rambaut A. Drummond A. Xie D. (2018). Tracerv1.7. Available online at: http://beast.community/tracer (accessed March 10, 2020).

[B48] Ramírez J. D. Flórez C. Olivera M. Bernal M. C. Giraldo J. C. (2017). *Blastocystis* subtyping and its association with intestinal parasites in children from different geographical regions of Colombia. PLoS ONE 12:e0172586. 10.1371/journal.pone.0172586 28222192 PMC5319748

[B49] Ramírez J. D. Sánchez A. Hernández C. Flórez C. Bernal M. C. Giraldo J. C.. (2016). Geographic distribution of human *Blastocystis* subtypes in South America. Infect. Genet. Evol. 41, 32–35. 10.1016/j.meegid.2016.03.017 27034056

[B50] Ramírez J. D. Sánchez L. V. Bautista D. C. Corredor A. F. Flórez A. C. Stensvold C. R. (2014). *Blastocystis* subtypes detected in humans and animals from Colombia. Infect. Genet. Evol. 22, 223–228. 10.1016/j.meegid.2013.07.020 23886615

[B51] Rezaei Riabi T. Haghighi A. Mirjalali H. Poirier P. Zali M. R. Delbac F.. (2018). Genetic diversity analysis of *Blastocystis* subtypes from both symptomatic and asymptomatic subjects using a barcoding region from the 18S rRNA gene. Infect. Genet. Evol. 61, 119–126. 10.1016/j.meegid.2018.03.026 29608961

[B52] Roberts T. Stark D. Harkness J. Ellis J. (2013). Subtype distribution of *Blastocystis* isolates from a variety of animals from New South Wales, Australia. Vet. Parasitol. 196, 85–89. 10.1016/j.vetpar.2013.01.011 23398989

[B53] Ronquist F. Teslenko M. Van der Mark P. Ayres D. L. Darling A. Höhna S.. (2012). Efficient bayesian phylogenetic inference and model choice across a large model space. Syst. Biol. 61, 539–542. 10.1093/sysbio/sys029 22357727 PMC3329765

[B54] Rossen N. G. Bart A. Verhaar N. van Nood E. Kootte R. de Groot P. F.. (2015). Low prevalence of *Blastocystis* sp. in active ulcerative colitis patients. Eur. J. Clin. Microbiol. Infect. Dis. 34, 1039–1044. 10.1007/s10096-015-2312-2 25680316 PMC4409634

[B55] Rudzińska M. Kowalewska B. Waz P. Sikorska K. Szostakowska B. (2019). *Blastocystis* subtypes isolated from travelers and non-travelers from the north of Poland—a single center study. Infect. Genet. Evol. 75, 10–26. 10.1016/j.meegid.2019.103926 31220611

[B56] Scanlan P. D. Knight R. Song S. J. Ackermann G. Cotter P. D. (2016). Prevalence and genetic diversity of *Blastocystis* in family units living in the United States. Infect. Genet. Evol. 45, 95–97. 10.1016/j.meegid.2016.08.018 27545648

[B57] Scanlan P. D. Marchesi J. R. (2008). Micro-eukaryotic diversity of the human distal gut microbiota: qualitative assessment using culture-dependent and -independent analysis of faeces. ISME J. 2, 1183–1193. 10.1038/ismej.2008.76 18670396

[B58] Scanlan P. D. Ross R. P. Stanton C. Cotter P. D. Hill C. J. Ryan C. A. (2018). The intestinal protist *Blastocystis* is not a common member of the healthy infant gut microbiota in a Westernized country (Ireland). Parasitol 145, 1274–1278. 10.1017/S0031182018000033 29397054

[B59] Scanlan P. D. Stensvold C. R. (2013). *Blastocystis*: getting to grips with our guileful guest. Trends Parasitol. 29, 523–529. 10.1016/j.pt.2013.08.006 24080063

[B60] Scanlan P. D. Stensvold C. R. Rajilić-Stojanović M. Heilig H. G. H. J. De Vos W. M. O'Toole P. W.. (2014). The microbial eukaryote *Blastocystis* is a prevalent and diverse member of the healthy human gut microbiota. FEMS Microbiol. Ecol. 90, 326–330. 10.1111/1574-6941.12396 25077936

[B61] Scicluna S. M. Tawari B. Clark C. G. (2006). DNA barcoding of *Blastocystis*. Protist 157, 77–85. 10.1016/j.protis.2005.12.001 16431158

[B62] Sekar U. Shanthi M. (2015). Recent insights into the genetic diversity, epidemiology and clinical relevance of *Blastocystis* species. J. Med. Res. 1, 33–39.

[B63] Seyer A. Karasartova D. Ruh E. Güreser A. S. Turgal E. Imir T.. (2017). Epidemiology and prevalence of *Blastocystis* spp. in North Cyprus. Am. J. Trop. Med. Hyg. 96, 1164–1170. 10.4269/ajtmh.16-0706 28167596 PMC5417212

[B64] Silberman J. D. Sogin M. L. Leipe D. D. Clark C. G. (1996). Human parasite finds taxonomic home. Nature 4, 380–398. 10.1038/380398a0 8602239

[B65] Stensvold C. R. Alfellani M. Clark C. G. (2012). Levels of genetic diversity vary dramatically between *Blastocystis* subtypes. Infect. Genet. Evol. 12, 263–273. 10.1016/j.meegid.2011.11.002 22116021

[B66] Stensvold C. R. Arendrup M. C. Jespersgaard C. Molbak K. Nielsen H. V. (2007). Detecting *Blastocystis* using parasitologic and DNA-based methods: a comparative study. Diag. Microbiol. Infect. Dis. 59, 303–307. 10.1016/j.diagmicrobio.2007.06.003 17913433

[B67] Stensvold C. R. Clark C. G. (2016a). Current status of *Blastocystis*: a personal view. Parasitol. Int. 65, 763–771. 10.1016/j.parint.2016.05.015 27247124

[B68] Stensvold C. R. Clark C. G. (2016b). Molecular identification and subtype analysis of *Blastocystis*. Curr. Protoc. Microbiol. 43, 1934–8525. 10.1002/cpmc.17 27858971

[B69] Stensvold C. R. Clark C. G. (2020). Pre-empting pandora's box: *Blastocystis* subtypes revisited. Trends Parasitol. 36, 229–232. 10.1016/j.pt.2019.12.009 32001133

[B70] Stensvold C. R. Lebbad M. Hansen A. Beser J. Belkessa S. Andersen L. O.. (2020b). Differentiation of *Blastocystis* and parasitic archamoebids encountered in untreated wastewater samples by amplicon-based next-generation sequencing. Parasite Epidemiol. Control. 9:e00131. 10.1016/j.parepi.2019.e00131 31909230 PMC6940715

[B71] Stensvold C. R. Lewis H. C. Hammerum A. M. Porsbo L. J. Nielsen S. S. Olsen K. E.. (2009). *Blastocystis*: unravelling potential risk factors and clinical significance of a common but neglected parasite. Epidemiol. Infect. 137, 1655–1663. 10.1017/S0950268809002672 19393117

[B72] Stensvold C. R. Tan K. S. W. Clark C. G. (2020a). *Blastocystis*. Trends Parasitol. 36, 315–316. 10.1016/j.pt.2019.12.008 32001134

[B73] Stensvold C. R. van der Giezen M. (2018). Associations between gut microbiota and common luminal intestinal parasites. Trends Parasitol. 34, 369–377. 10.1016/j.pt.2018.02.004 29567298

[B74] Suresh K. Smith H. (2004). Comparison of methods for detecting *Blastocystis hominis*. Eur. J. Clin. Microbiol. Infect. Dis. 23, 509–511. 10.1007/s10096-004-1123-7 15168139

[B75] Tito R. Y. Chaffron S. Caenepeel C. Lima-Mendez G. Wang J. Vieira-Silva S.. (2019). Population-level analysis of *Blastocystis* subtype prevalence and variation in the human gut microbiota. Gut 68, 1180–1189. 10.1136/gutjnl-2018-316106 30171064 PMC6582744

[B76] Trifinopoulos J. Nguyen L. T. von Haeseler A. Minh B. Q. (2016). W-IQ-TREE: a fastonline phylogenetic tool for maximum likelihood analysis. Nucleic Acids Res. 44, 232–235. 10.1093/nar/gkw256 27084950 PMC4987875

[B77] Udonsom R. Changbunjong T. Mori H. Popruk S. Mahittikorn A. Prasertbun R.. (2018). *Blastocystis* infection and subtype distribution in humans, cattle, goats, and pigs in central and western Thailand. Infect. Genet. Evol. 65, 107–111. 10.1016/j.meegid.2018.07.007 30003970

[B78] Valença Barbosa C. De Jesus Batista R. Pereira Igreja R. D'Avila Levy C. M. Werneck De Macedo H.. (2017). Distribution of *Blastocystis* subtypes isolated from humans from an urban community in Rio de Janeiro, Brazil. Parasit. Vectors 10:518. 10.1186/s13071-017-2458-0 29070053 PMC5657060

[B79] Valença-Barbosa C. Do Bomfim T. C. B. Teixeira B. R. Gentile R. Da Costa Neto S. F. Magalhães B. S. N.. (2019). Molecular epidemiology of *Blastocystis* isolated from animals in the state of Rio de Janeiro, Brazil. PLoS ONE 14:e0210740. 10.1371/journal.pone.0210740 30682075 PMC6347289

[B80] van Hattem J. M. Arcilla M. S. Schultsz C. Bootsma M. C. Verhaar N. Rebers S. P.. (2019). Carriage of *Blastocystis* spp. in travellers - A prospective longitudinal study. Travel Med. Infect. Dis. 27, 87–91. 10.1016/j.tmaid.2018.06.005 29929001

[B81] Wang J. Gong B. Liu X. Zhao W. Bu T. Zhang W.. (2018). Distribution and genetic diversity of *Blastocystis* subtypes in various mammal and bird species in northeastern China. Parasit. Vectors 11:522. 10.1186/s13071-018-3106-z 30236147 PMC6148767

[B82] Wang W. Cuttell L. Bielefeldt-Ohmann H. Inpankaew T. Owen H. Traub R. J. (2013). Diversity of *Blastocystis* subtypes in dogs in different geographical settings. Parasit. Vectors 24, 215–220. 10.1186/1756-3305-6-215 23883734 PMC3734043

[B83] Wang W. Owen H. Traub R. J. Cuttell L. Inpankaew T. Bielefeldt-Ohmann H. (2014). Molecular epidemiology of *Blastocystis* in pigs and their in-contact humans in Southeast Queensland, Australia, and Cambodia. Vet. Parasitol. 203, 264–269. 10.1016/j.vetpar.2014.04.006 24785292

[B84] Wawrzyniak I. Poirier P. Texier C. Delbac F. Viscogliosi E. Dionigia M.. (2013). Distribution and genetic diversity of *Blastocystis* subtypes in various mammal and bird species in northeastern China. Ther. Adv. Infect. Dis. 1, 167–178. 10.1177/2049936113504754 25165551 PMC4040727

[B85] Yan Y. Su S. Ye J. Lai X. Lai R. Liao H.. (2007). *Blastocystis* sp. subtype 5: a possibly zoonotic genotype. Parasitol. Res. 101, 1527–1532. 10.1007/s00436-007-0672-y 17665214

[B86] Yoshikawa H. Koyama Y. Tsuchiya E. Takami K. (2016). *Blastocystis* phylogeny among various isolates from humans to insects. Parasitol. Int. 65, 750–759. 10.1016/j.parint.2016.04.004 27091546

[B87] Zaman V. Ng G. C. Suresh K. Yap E. H. Sing M. (1993). Isolation of *Blastocystis* from the cockroach (*Diptera*:*Blattidae*). Parasitol. Res. 79, 73–74. 10.1007/BF00931221

