# Supplementary figures and images for: A Study on the Prevalence and Subtype Diversity of the Intestinal Protist Blastocystis sp. in a Gut-Healthy Human Population in the Czech Republic
Source: Front Cell Infect Microbiol. 2020 Oct 6;10:544335. doi: 10.3389/fcimb.2020.544335 (PMC7573152; doi:10.3389/fcimb.2020.544335)

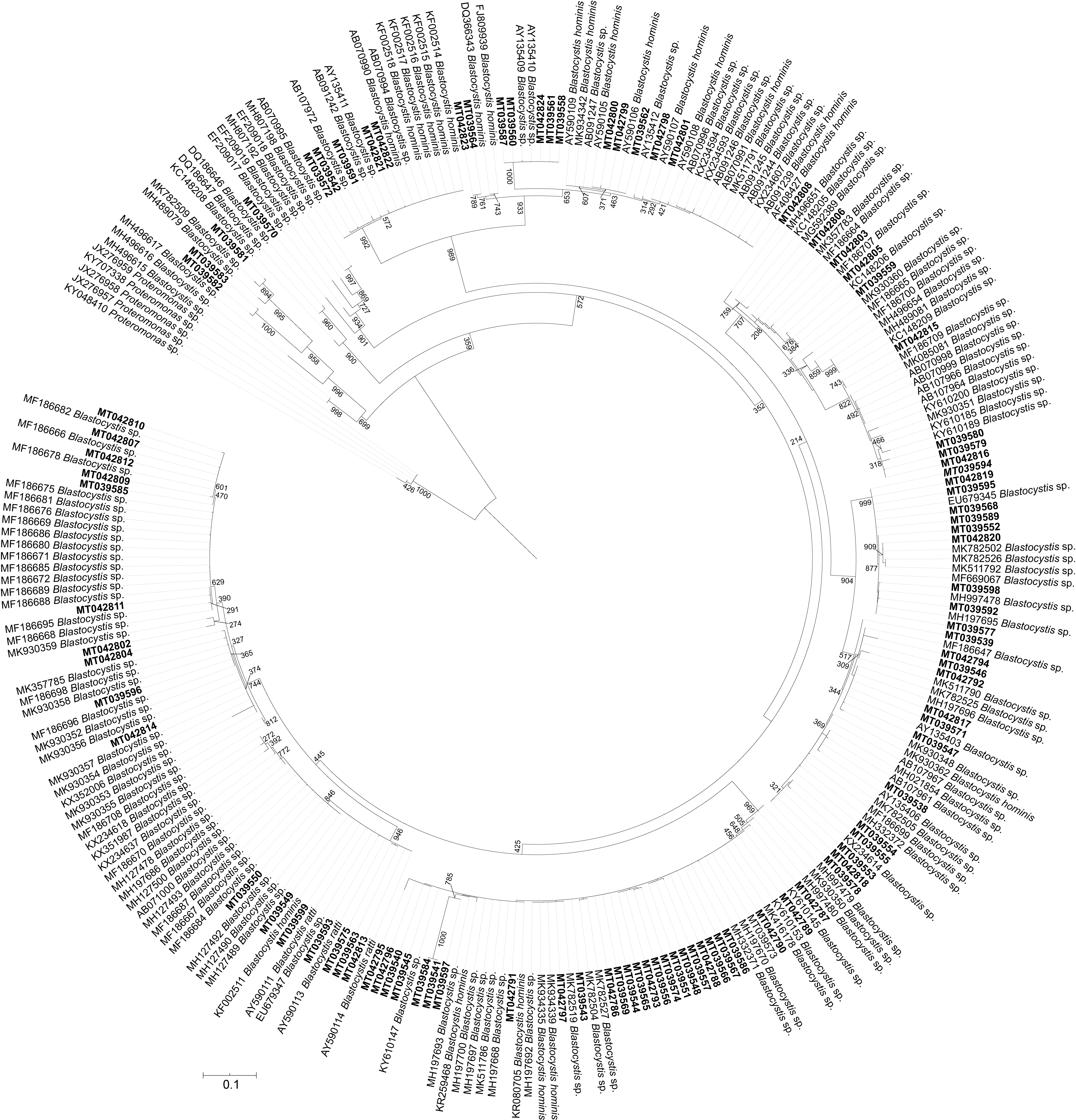

Supplement: Supplementary Material 3 — The ML tree based on Blastocystis SSU rDNA sequences, statistical support for each branch was obtained from maximum-likelihood bootstrap; supports lower than 200 are not shown. Sequences of Proteromonas sp. were used as an outgroup to root the presented tree; data obtained for this study are highlighted by bold. [file Image_1.TIF]
